# Genetic structure and geneflow of *Malus* across the Korean Peninsula using genotyping-by-sequencing

**DOI:** 10.1038/s41598-022-20513-z

**Published:** 2022-09-28

**Authors:** Young-Ho Ha, Hee-Young Gil, Sang-Chul Kim, Kyung Choi, Joo-Hwan Kim

**Affiliations:** 1 Division of Forest Biodiversity, Korea National Arboretum, Pocheon, Gyeonggi-do 11186 Republic of Korea; 2 Department of Life Science, Gachon University, Seongnam, Gyeonggi-do 13120 Republic of Korea

**Keywords:** Evolutionary biology, Genetic hybridization, Genetic markers, Genotype, Plant genetics

## Abstract

This study was to understand the genetic structure and diversity of the Korean *Malus* species. We used genotyping-by-sequencing (GBS) technology to analyze samples of 112 individuals belonging to 18 populations of wild *Malus* spp. Using GBS, we identified thousands of single nucleotide polymorphisms in the species analyzed. *M. baccata* and *M. toringo*, two dominant mainland species of the Korean Peninsula, were distinguishable based on their genetic structure. However, *M. toringo* collected from Jeju Island exhibited a different genetic profile than that from the mainland. We identified *M.* cf. *micromalus* as a hybrid resulting from the Jeju Island *M. toringo* (pollen donor) and the mainland *M. baccata*, (pollen recipient). Putative *M. mandshurica* distributed on the Korean Peninsula showed a high structural and genetic similarity with *M. baccata*, indicating that it might be an ecotype. Overall, this study contributes to the understanding of the population history and genetic structure of *Malus* in the Korean Peninsula.

## Introduction

*Malus* Mill. (tribe Maleae, Rosaceae) is an economically important genus comprising 25–55 taxa and is widely distributed in the temperate regions of the northern hemisphere^1–3^. *Malus domestica* Borkh., a representative crop of apples, underwent hybridization and gene introgression with various wild *Malus* species over decades^4^. Interspecific hybridization in *Malus* is well documented^5–9^. Although several taxonomists proposed reasonable classification systems based on its morphology^10–19^, reticulated processes of *Malus* have hindered the accuracy of its phylogenetic inferences^5–7^. The concept of hybridization leading to the formation of new species has been proposed in the 1950s^20^. Hybrids usually possess an intermediate phenotype of the two parental species; describing their morphology can be subjective and difficult to predict owing to the combined expression of parental genes^21^. Some hybrids are identified based on their morphological properties, but their origins remain unknown (e.g., *Malus* × *robusta* Rehd. and *Malus* × *micromalus* Mak.; assessed at 30th April 2022 https://www.treesandshrubsonline.org/). Similarly, distinguishing interspecific hybrids of *Malus* among species of the Korean Peninsula can be difficult. Since the identification of two subspecies by Nakai^10^ (*Pyrus baccata* var. *sibirica* Maxim. [synonym of *Malus rockii* Rehder] and *Pyrus baccata* var. *manshurica* Maxim. [synonym of *Malus manshurica* (Maxim.) Kom. ex Skvortsov]), up to eight taxa including cultivar have been reported in the Korean Peninsula over the last 20 years (Table S1). *M. baccata* is primarily distributed in northern Asia (Bhutan, China, India, Kashmir, Korea, Mongolia, Nepal, and Russia) and has been widely used as a rootstock for breeding because it is disease-free and cold-resistant^22–24^.

**Figure 1:**
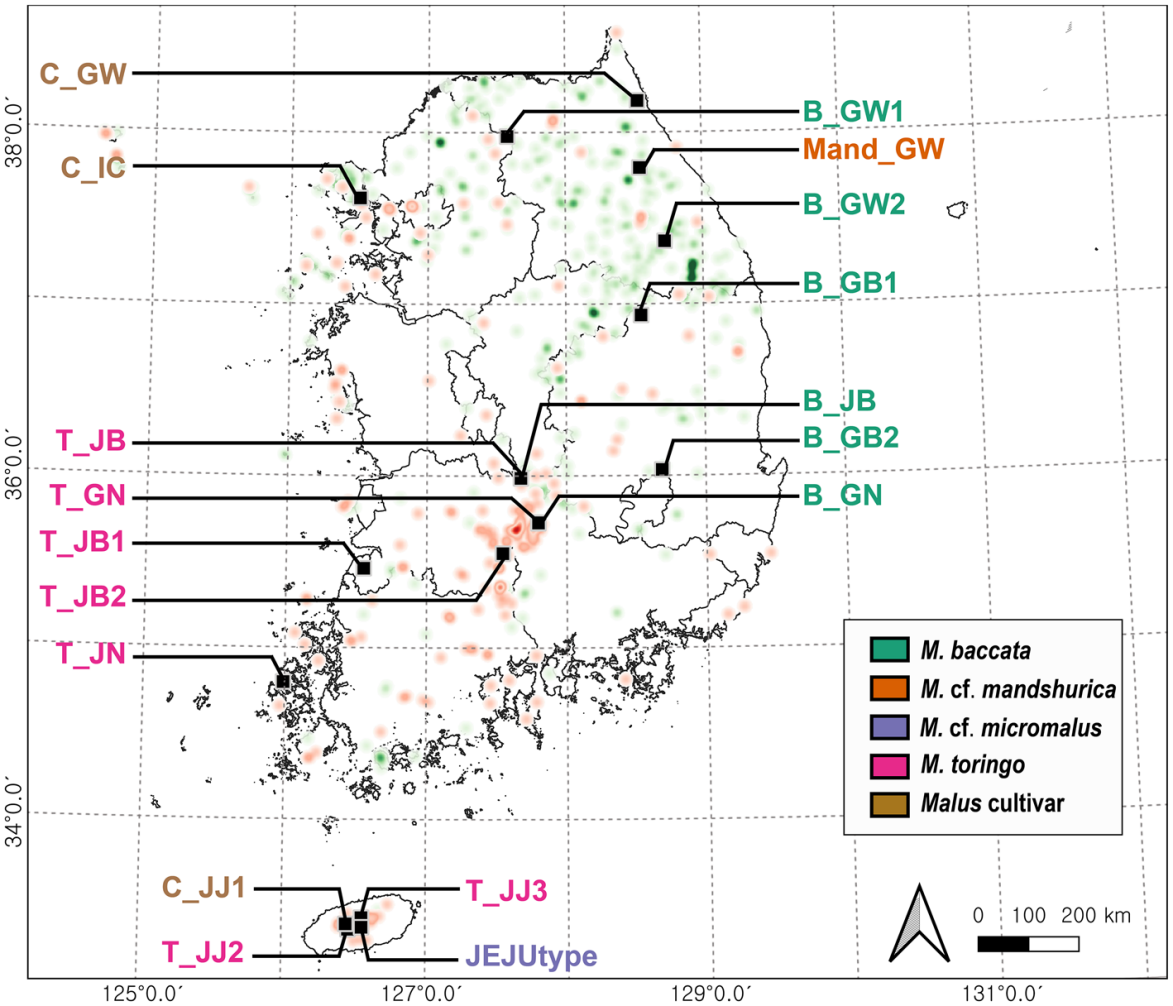
Heat map of 833 specimens deposited in both the Korea National Arboretum Herbarium (KH) and the National Institute of Biological Resources Herbarium (KB) were visualized using Qgis v3.26.1. Red color indicates *M. toringo* and green represents *M. baccata*. The black square indicates the sampling sites information for this study. Map source: https://www.gisdeveloper.co.kr/.

*M. baccata* and *M*. *toringo* (Siebold) Siebold ex de Vriese are the most widely distributed representative taxa in the Korean Peninsula^10–19^. *M. baccata* is predominantly distributed along the main mountain-range connecting the north and the south (called Baekdu-daegan) up to the central region and is vertically distributed from the lowlands to about 1500 m above sea level (Fig. 1). *M. toringo,* a 2–6 m small ornamental tree or shrub, is distributed in East Asian countries including China, Japan, Korea, and Russia^25^. In the Korean Peninsula, *M. toringo* is distributed in the central and central-southern regions and in the Jeju Island mainly growing in lowland areas (Fig. 1)^11,12^.

*M. baccata* and *M. toringo* can be morphologically differentiated based on their leaf lobes (*M. baccata*: not lobed and *M. toringo*: 3–5 lobed leaf), petiole (*M. baccata*: 2–5 cm and *M. toringo*: 1.5–2.5 cm), pedicel length (*M. baccata*: 4–7 cm and *M. toringo*: 1.2–4 cm), bud characteristics (*M. baccata* lineage: convoluted bud and *M. toringo* lineage: folded bud), and fruit size (*M. baccata*: 6–9 mm and *M. toringo*: 4–6 mm)^11–14,25,26^. However, individuals with intermediate morphology between *M. baccata* and *M. toringo* possess the following characteristics^27^: (1) ovate leaf but slightly lobed, short petiole and pedicel; (2) slightly lobed leaf, long petiole and pedicel; and (3) variations in fruit sizes.

Previous research has not deduced consistent results with regard to the relationship between the two species. Although phylogenetic studies performed using sequences of partial chloroplast and nuclear regions have confirmed that *M. baccata* and *M. toringo* are distinguished based on their morphological characters^28,29^, maximum likelihood (ML) tree constructed using 79 protein-coding chloroplast genes clustered the sequences based on geographic distribution, and not the species monophyletic group^30^. In addition, the unweighted pair group method with arithmetic mean (UPGMA), based on morphological characters and random amplified polymorphic DNA (RAPD) analysis, revealed that the individuals formed two distinct clusters based on species data^31,32^. Intermediate morphological features^14,27^ and inconsistent molecular evidence^30^ have raised doubts over the natural hybridization of the two species as suggested by Chang et al.^14^; however, no assessment has been conducted at the population level.

*M. micromalus* Makino is another putative hybrid species, which was first described by Makino in 1908, and introduced in South Korea from China^33^. This species is presumed to have resulted from a cross between *M. baccata* and *M. spectabilis* (Sol.) Borkh^25,34^. *M. micromalus* is distributed at high elevations in areas such as the Hallasan Mountain (> 1500 m) on Jeju Island, which is approximately 140 km south of the Korean Peninsula mainland^33^. The species has the following morphological characteristics: tomentose in the midrib, pubescent petiole when young, pedicels tomentose-pubescent, and persistent calyx at pomes^10–18,33^. Although this taxon has been described in various studies, there are discrepancies in its classification^12,13^. Moreover, literature on whether calyx is caducous or persistent is inconsistent^11,15,18,33^, making it difficult to identify distinct morphological differences between *M. micromalus* and other related species.

Another relevant species is *M. mandshurica* (Maxim.) Kom. ex Juz., which is distributed in China, Russia, Japan, North Korea, and South Korea^25^. Although *M. mandshurica* possess similar characteristics as *M. baccata*, it exhibits sparse pubescence on the petiole and abaxial pubescence on leaves, or subglabrous along midvein and lateral veins^25,26^. However, preliminary investigation of specimen and image data of both herbariums (Herbarium of Korea National Arboretum: KH; National Institute of Biological Resources: KB) revealed that it was difficult to differentiate *M. mandshurica* from *M. baccata*. Moreover, there have been discrepancies in the ranking of this taxon, with some studies classifying it as a separate species and others classifying it as a variety of *M. baccata*^16,19,25,26^. Additionally, *M. mandshurica* has also been classified as an ecotype and treated as a synonym of *M. baccata*^14,35^. Previous studies that performed simple sequence repeat (SSR) analyses using several markers could not differentiate the two species because the genetic distance within *M. baccata* taxa was scattered across other *Malus* species^36^. Additionally, the low resolution of a phylogenetic approach cannot efficiently represent the relationship between the two species^29,32,37^. Species boundaries are often difficult to identify morphologically; therefore, integrated taxonomy which includes additional information, such as molecular data, biogeography, and ecology is required^38,39^. When several lines of evidence (e.g., morphology, molecules, ecology, or distribution) independently indicate a certain species boundary, discretely evolving lineages can be identified^40–42^.

Recently, next-generation sequencing (NGS) has identified sufficient single nucleotide polymorphisms (SNPs) from population genetic studies to facilitate the study of genetic diversity in *Malus*^43–45^. Molecular diagnosis of genetic diversity within the nuclear genome using GBS has the potential to examine hybrid signals, introgressions, and the population–species boundary^46–48^. This is the first study to explore the possibility of hybridization and evolutionary relationship of *Malus* across the Korean Peninsula. The aim of this study was to identify *Malus-*specific SNPs in the Korean Peninsula and establish their phylogenetic relationships by GBS approaches. The objectives of this study were to (1) verify whether there is a hybrid or genetic exchange between *M. baccata* and *M. toringo*; (2) identify the entity of *M*. cf. *micromalus* distributed in Jeju Island; (3) compare the genetic structure of *M. toringo* distributed in the mainland and Jeju Island; and (4) verify the entity of *M.* cf. *madshurica* collected in the Korean Peninsula.

## Methods

### Sample collection and DNA extraction

In this study, we collected a total of 112 individuals (5 taxa 18 populations) from natural habitats (Table S2) as well as Sequence Read Archive (SRA) data (Table S3) from the National Center for Biotechnology Information (NCBI). A total of 25 accessions of SRA data (10 accessions of *M. baccata*, three accessions of *M. toringo*, four accessions of *M. mandshurica*, four accessions of *M. micromlaus*, and three accessions of *M. spectabilis*) were obtained from a previously conducted GBS study^49^. During the field survey, young leaves were collected from 112 individuals belonging to 18 populations (six populations of *M. baccata*, seven populations of *M. toringo*, one population of *M. mandshurica*, one population of *M.* cf. *micromalus*, and three populations of *Malus* sp. cultivar) in South Korea (Table 1, Fig. 1). The location of the collected samples was 30 km away from each other in the mainland and 10 km away from each other in the Jeju Island (73 km east–west and 31 km north–south). Total genomic DNA was extracted from silica-dried plant material using a DNeasy Plant Mini Kit (Qiagen, Valencia, CA, USA), following the manufacturer’s instructions. All voucher specimens were deposited in the KH (detail of voucher number is listed in S2).

**Table 1:** Measures of diversity for 112 *Malus* accessions from five 18 populations calculated from 8426 SNPs. *PG*, population group; *P*, private alleles, *N*, number of individuals; *Ho*, observed heterozygosity; *He*, expected heterozygosity; Pi (π), nucleotide diversity; *F*_*IS*_, inbreeding coefficient.

Scientific name	Pop ID	PG	P	N	*Ho*	*He*	Pi (π)	*F*_*IS*_
*M. baccata*	B_GW1	C1	0	5	0.14	0.10	0.11	− 0.05
*M. baccata*	Baccata_GW2	C1	0	5	0.14	0.11	0.13	− 0.03
*M. baccata*	Baccata_GB1	C1	0	5	0.13	0.11	0.13	0.00
*M. baccata*	Baccata_GB2	C2	0	5	0.25	0.14	0.16	− 0.17
*M. baccata*	Baccata_JB	C2	0	13	0.24	0.13	0.15	− 0.16
*M. baccata*	Baccata_GN	C2	0	3	0.24	0.16	0.18	− 0.12
*M. toringo*	Toringo_JB1	C3	0	10	0.14	0.08	0.09	− 0.11
*M. toringo*	Toringo_JB2	C3	0	5	0.14	0.08	0.09	− 0.11
*M. toringo*	Toringo_JB	C3	0	5	0.14	0.08	0.09	− 0.10
*M. toringo*	Toringo_GN	C3	0	5	0.14	0.08	0.08	− 0.10
*M. toringo*	Toringo_JN	C3	0	4	0.15	0.08	0.08	− 0.12
*Malus* cultivar	Cultivar_GW	C4	0	5	0.07	0.07	0.08	0.01
*Malus* cultivar	Cultivar_JJ1	C4	0	4	0.09	0.09	0.10	0.02
*Malus* cultivar	Cultivar_IC	C4	0	5	0.15	0.12	0.15	− 0.01
*M. toringo*	Toringo_JJ2	C5	1	5	0.13	0.13	0.13	0.01
*M. toringo*	Toringo_JJ3	C5	0	5	0.12	0.11	0.12	0.01
*M.* cf*. micromalus*	JEJUtype	C6	6	13	0.21	0.19	0.20	− 0.02
*M.* cf*. mandshurica*	Mand_GW	C7	1	10	0.12	0.13	0.14	0.04

### GBS library construction and NGS

The GBS libraries of 112 individuals were constructed as previously described^50^, with minor modifications. Briefly, DNA samples were digested with *Ape*KI (New England Biolabs, Ipswich, MA, USA). Adapters were subsequently ligated to the sticky ends by adding T4 DNA ligase (200 U; MGMED, Korea) to each well. Digested DNA samples, each with a different barcode adapter, were combined and purified using a commercial kit (QIAquick PCR Purification Kit; Qiagen, USA), according to the manufacturer’s instructions. The libraries were sequenced using Illumina NextSeq500 for 95 samples and Hi-seqX for 20 samples.

### Mapping to reference genome and SNP calling

After sequencing, raw reads were de-multiplexed according to the barcode sequences using the “process_radtags” function in STACKS v2.60 with default parameters: “—inline-null” for barcode option and “-e apeKI” for enzymes option^51^. Reads were trimmed, and adaptors were removed using cutadapt 3.5^52^. In this study, two data matrices were used: (1) data set A: SRA data (25 accessions) + 112 individuals of *Malus* species collected from the Korean Peninsula; (2) data set B: 112 individuals of *Malus* species. A total of 137 individual reads were aligned to the *Malus domestica* chromosome sequence retrieved from NCBI (ASM211411v1) to generate the BAM files using BWA v0.7.17 and SAMtools v1.9 with the default parameters^53–55^ (Tables S2 and S3). The generated BAM files were input into the gstacks, a core program included in STACKS v2.60, under default parameters.

Two major output files were generated (catalog.fa.gz and catalog.calls) which were subsequently input into the “Populations” program of STACKS v2.60 that utilize SNP calling. The ‘-p’ option, which indicates the minimum number of populations required to process a locus, was set to 23 for 137 accessions (data set A) and 18 for 112 accessions (data set B). The designation of parameters 23 and 18 in two data sets for SNPs calling is a strict strategy to extract only the SNPs observed in all groups. The minimum percentage of individuals in a population required to process a locus for that population (-r) was set to 1 for 137 accessions of data set A because for the maximum value (1) is to minimize the error of SNPs generated due to external data. And we set the 0.8 for 112 accessions of data set B. The options listed were later equally applied to both datasets. The minimum percentage of individuals in/across a population required to process a locus for that population were increased (--min-samples-per-pop 1, --min-samples-overall 1). Additionally, we set the minimum minor allele frequency to 0.05 (--min_maf 0.05), maximum observed heterozygosity to 0.95 (--max_obs_het 0.95) and restricted the study to only the first SNP per locus (--write-single-snp).

### Distribution map

Qgis v3.26.1 desktop application (https://qgis.org/en/site/) was used for visualizing the heat map and the source of GPS coordinates was collected from 833 specimens deposited in KH and KB herbariums.

### SplitsTree analysis vs minimum spanning network

To explore the genetic distance of data set A and B, we constructed a SplitsTree network using strictly filtered 563 and 613 SNPs, respectively. The vcf file generated from “populations” in STACKS v2.60 was converted into the FASTA format from the vcf2phylip (https://github.com/edgardomortiz/vcf2phylip.git), with the value for minimum samples per locus (MIN_SAMPLES_LOCUS) set to 137 in data set A and 112 in data set B. The network was created and visualized using SplitsTree4 software^56^.

### Population genetic analysis

To investigate the population structure, we used VCF file generated using the “populations” function in STACKS v2.60. To illustrate the relationship among various individuals, principal component analysis (PCA) was conducted based on 563 SNPs (137 accession) in data set A and 8426 SNPs (112 accession) in data set B, using graph Laplacian PCA (gLPCA)^57^ and plotted using R studio^58^. To investigate the population structure, plink files were converted into BED files, using Plink v1.07^59^, which were then used as input files to determine cross validation (CV) of K = 1–10 values, using admixture_ linux-1.3.0^60^. The graphical display of the population structure was generated using DISTRUCT^61^.

### Genetic diversity and differentiation

Common measures of genetic diversity, including private allele number (AP), percentage of polymorphic loci (%Poly), observed and expected heterozygosity (*Ho* and *He*), nucleotide diversity (*π*), inbreeding coefficient (*F*_*IS*_), and population differentiation (pairwise *F*_*ST*_), were calculated using the “populations” function in STACKS v2.60.

An analysis of molecular variance (AMOVA) was performed to estimate genetic variation among and within populations, using the adegenet^62^ and poppr^63^ package in R studio^64^. The AMOVA analysis was performed for four categories: (1) all samples (C1–C7; 112 individuals); (2) *M. toringo*, including C3 and C5 (60 individuals); (3) *M. baccata* (C1 and C2) and *M. mandshurica* (C7; 39 individuals); (4) *M. baccata* (C1 and C2), *M. mandshurica* (C7), and *M. micromalus* (C6; 52 species), as well as *M. toringo* (C3 and C5) and *M. micromalus* (C6; 73 individuals).

### Migration rates calculation

To investigate the ancient gene flow, we utilized a coalescent approach implemented in MIGRATE- N 3.6.11 to calculate the migration rates between groups representing populations^65,66^. Structure file produced from the “populations” program of STACKS v2.60 was converted into the SNP model (heat map data) using the “vcfR2migrate” plug in vcfR package^67^. We redefined groups based on the genetic clusters inferred from STRUCTURE (Table 1). We set starting values of θ and M with an “Estimate with F_ST_ measure.” The gene flow parameter M was used (M = m/μ; m, immigration rate per generation; μ, mutation rate), with the mutation rate as a constant^68^. A more permissive acceptance criteria was applied (“heating”) with four chains set at different temperatures (1.0, 1.5, 3.0, and 100,000). The run used 100 long-samples with an increment of 10,000 (1,000,000 iterations) after a burn-in of 100,000. The number of migrants per generation (Nm) was calculated using the following equation: Nm = [(θ x * M y → x)/4])^68^.

### Ethics declarations

The materials used in this study are not included IUCN red list. Sample collections was conducted in compliance with the regulations of the Act on the creation and furtherance of arboretums and gardens.

## Results

### SNP discovery using data set A and B

A total of 397,876,068 raw reads (average of 3.5 million reads per sample) were generated from data set B (Table S2). After quality filtering, a total of 368,008,854 reads from 112 accessions and 46,503,805 reads from 25 accessions (five taxa, NCBI SRA data) were aligned to the *Malus domestica* reference genome. Approximately 83.2% of the 112 accessions and 92.3% of the 25 SRA data were mapped to the genome (Table S3). Subsequent analysis was performed using the generated BAM file, and SNP calling was performed with two separate data consisting of 112 accessions (data set B) collected from the Korean Peninsula and 137 accessions from data set A, including SRA data (Tables S2 and S3). Finally, a total of 562 SNPs were identified from 23 populations in data set A and 8426 SNPs were identified in data set B representing 18 Korean populations.

### Phylogenetic network relationship and genetic structure of data set A and B

**Figure 2:**
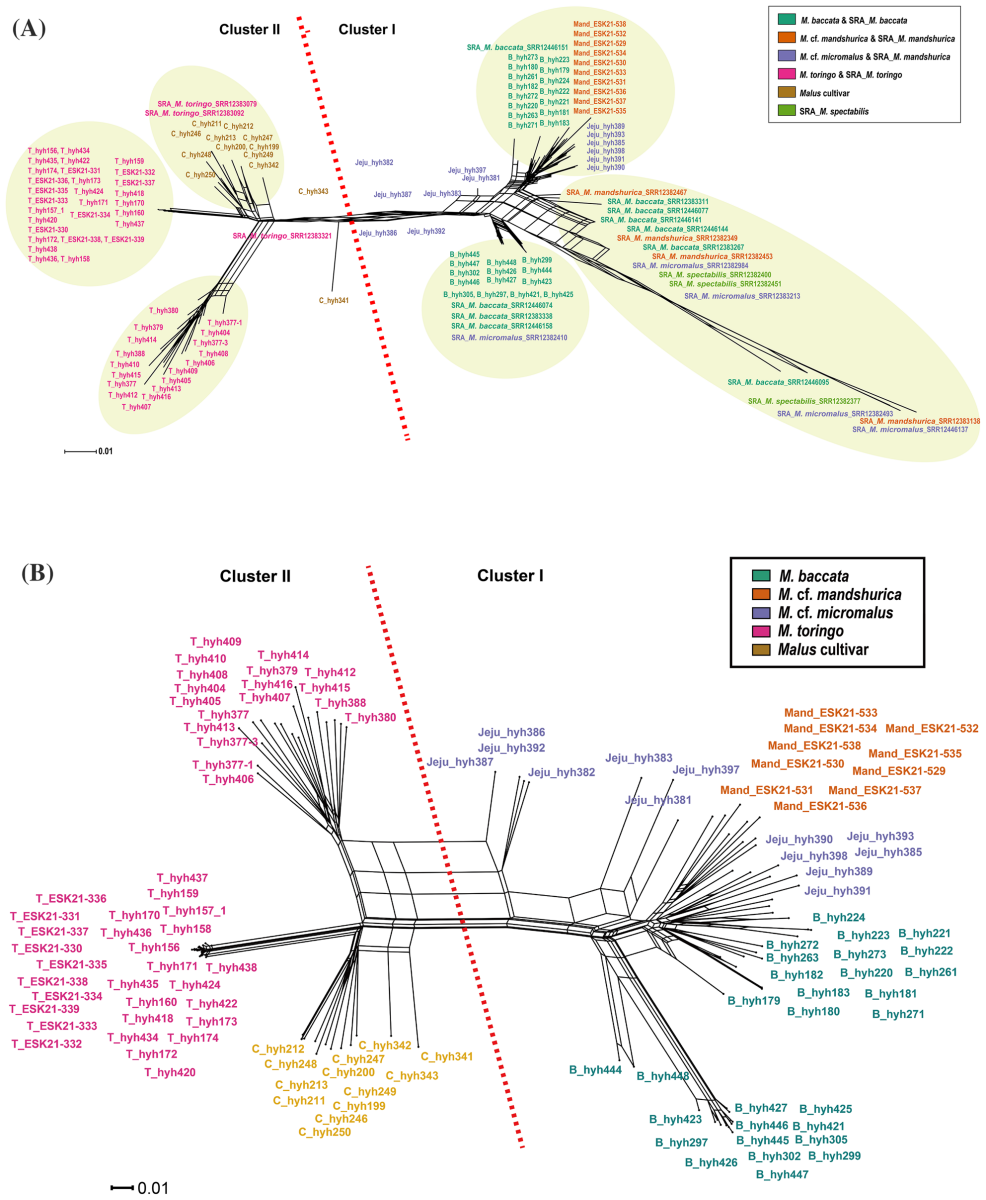
Unrooted network of 137 species of the *Malus* genus. (**A**) Neighbor-Net constructed by SplitsTree using 563 SNP markers of data set A. (**B**) Neighbor-Net constructed by SplitsTree using 612 SNP markers of data set B. Red dotted line indicates the division of 137 species based on the different leaf morphological characters (Cluster I: Leaf blade not lobed, and Cluster II: Leaf blade usually lobed). Capital in samples name indicate the abbreviation of scientific name (B: *M. baccata*, C: *Malus* cultivar, T: *M. toringo*, Ma: *M. mandshurica*, Mi: *M. micromalus*).

**Figure 3:**
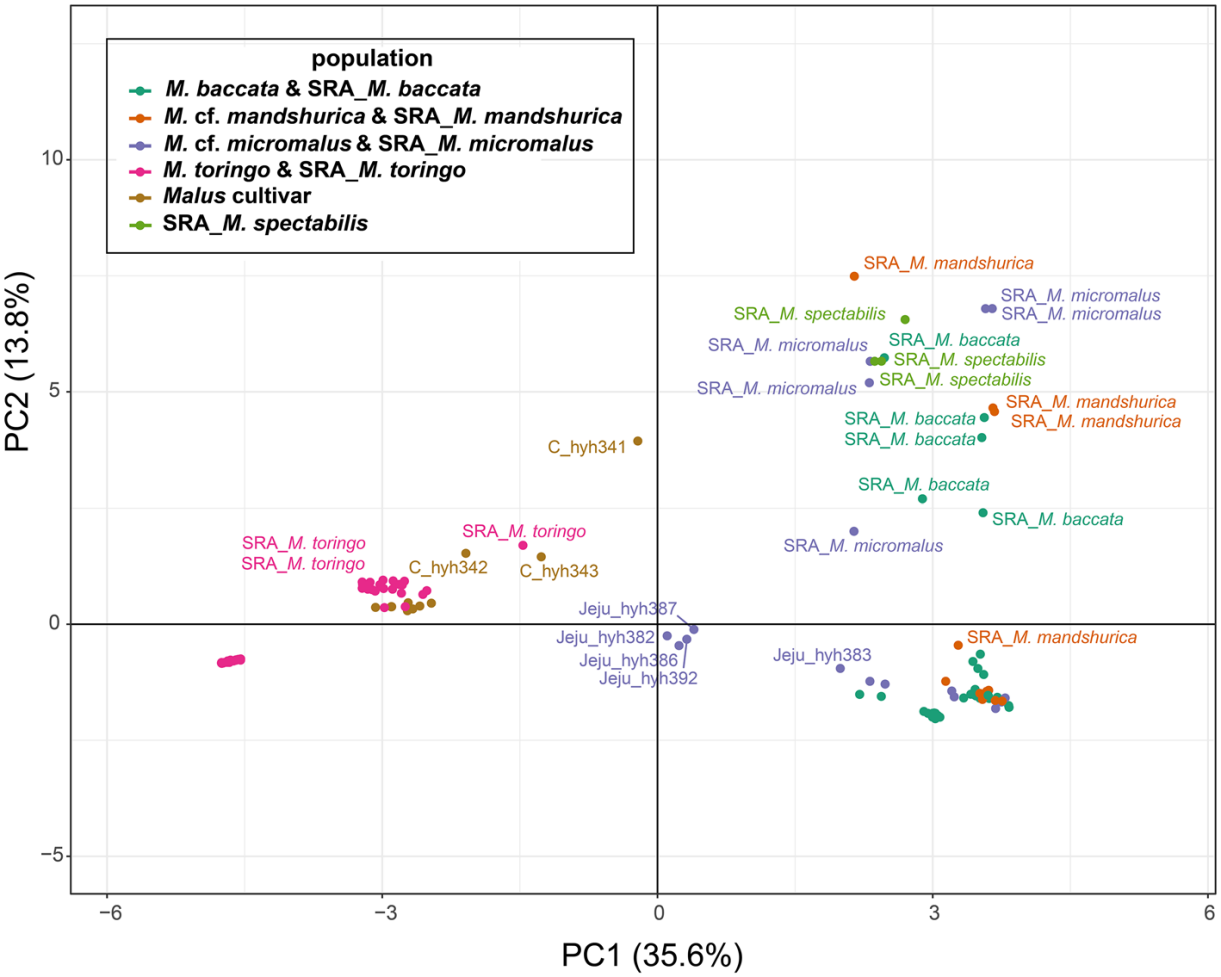
PCA of 137 *Malus* species was conducted with the top two components.

Phylogenetic networks were constructed to determine the relationships among *Malus* and its relatives (Fig. 2A,B). Both of the phylogenetic networks generated using data sets A and B were divided into two clusters (Fig. 2A,B). Cluster I in Fig. 2A included all accession of *M. baccata, M. micromalus* (including *M.* cf. *micromalus* in JEJU type population)*, M. mandshurica* (including *M.* cf. *mandshurica* in Mand_GW population), and *M. spectabilis.* Within cluster I, comparisons between collected samples and SRA data formed a subgroup based on their original collection site. Particularly, four SRA-*M. baccata* (SRR12446151: Korea_Wild_Apple; SRR12446074 and SRR12383338: China; SRR12446158: unkown^49^) were closely related to the Korean population (Fig. 2A). However, most of the SRA data of *M. mandshurica*, *M. micromalus, M. spectabilis,* and *M. baccata* (SRR12383267, 12383311, 12446095, 12446077, 12446141, and 12446144) were found to be of a different lineage, a long branch that was separately from the Korean population. Nevertheless, no clear distinction at the species level could be observed in the SRA branch. Putative *M. micromalus* collected from Jeju Island (hyh381-398; Table S2) formed separate clusters from SRA-*M. micromalus*, with some individuals related to *M. baccata*, while more than half were positioned at the border with cluster II. Similarly, *M. mandsurica* did not form a cluster with SRA-*M. mandsurica* data and *M.* cf. *mandsurica* collected from the Korean Peninsula. Furthermore, cluster II included all *M. toringo* accessions and three cultivar that maintained an independent group. Two separate groups were identified within the *M. toringo* accessions in SplitsTree. SRA-*M. toringo* was more similar to the cultivar than the Korean *M. toringo*. Additionally, PCA divided *M. toringo* into two groups: PC1 and PC2 (Fig. 3).

### Population genetic structure and relationship analysis using data set B

**Figure 4:**
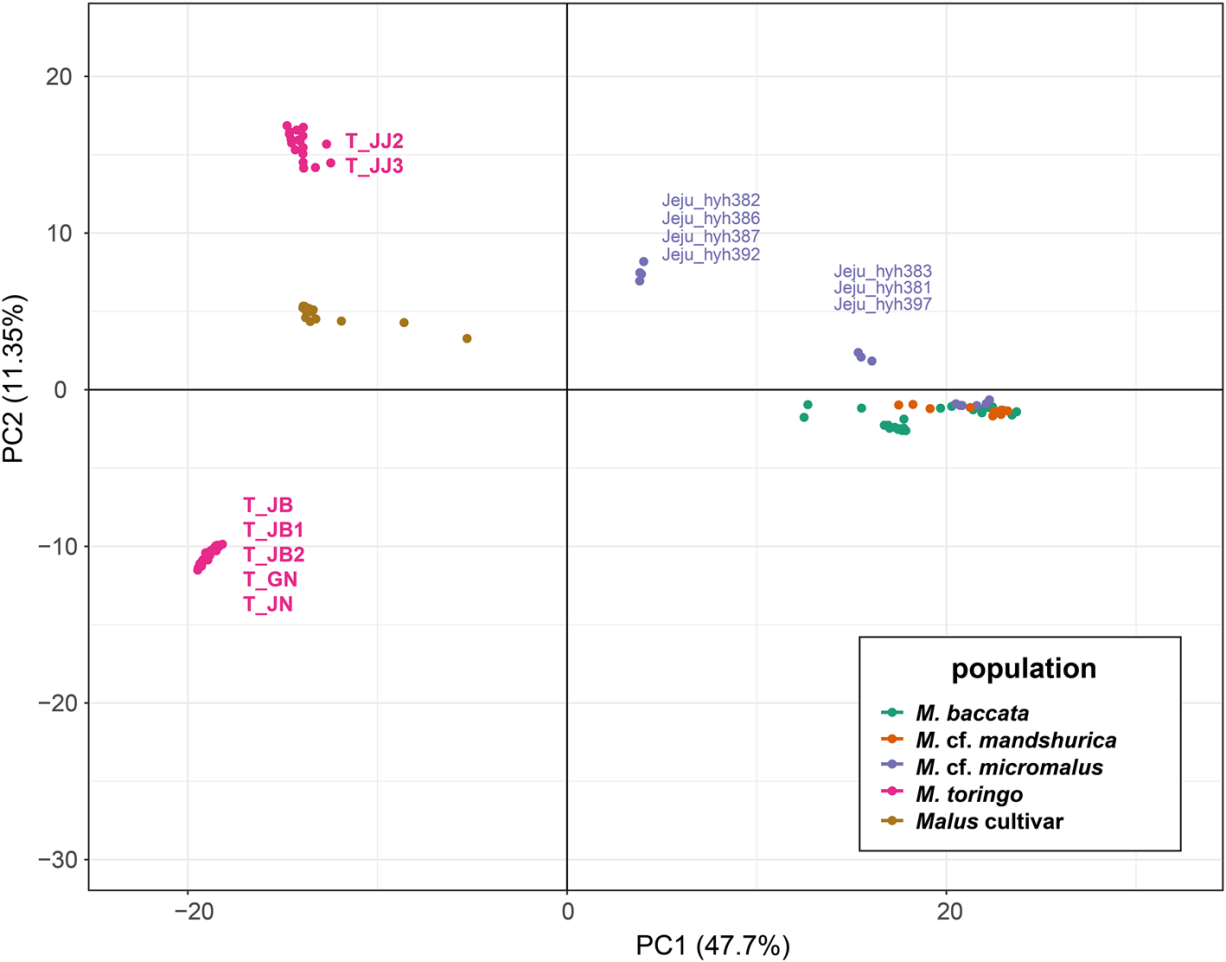
PCA of 112 *Malus* species was conducted with the top two components.

**Figure 5:**
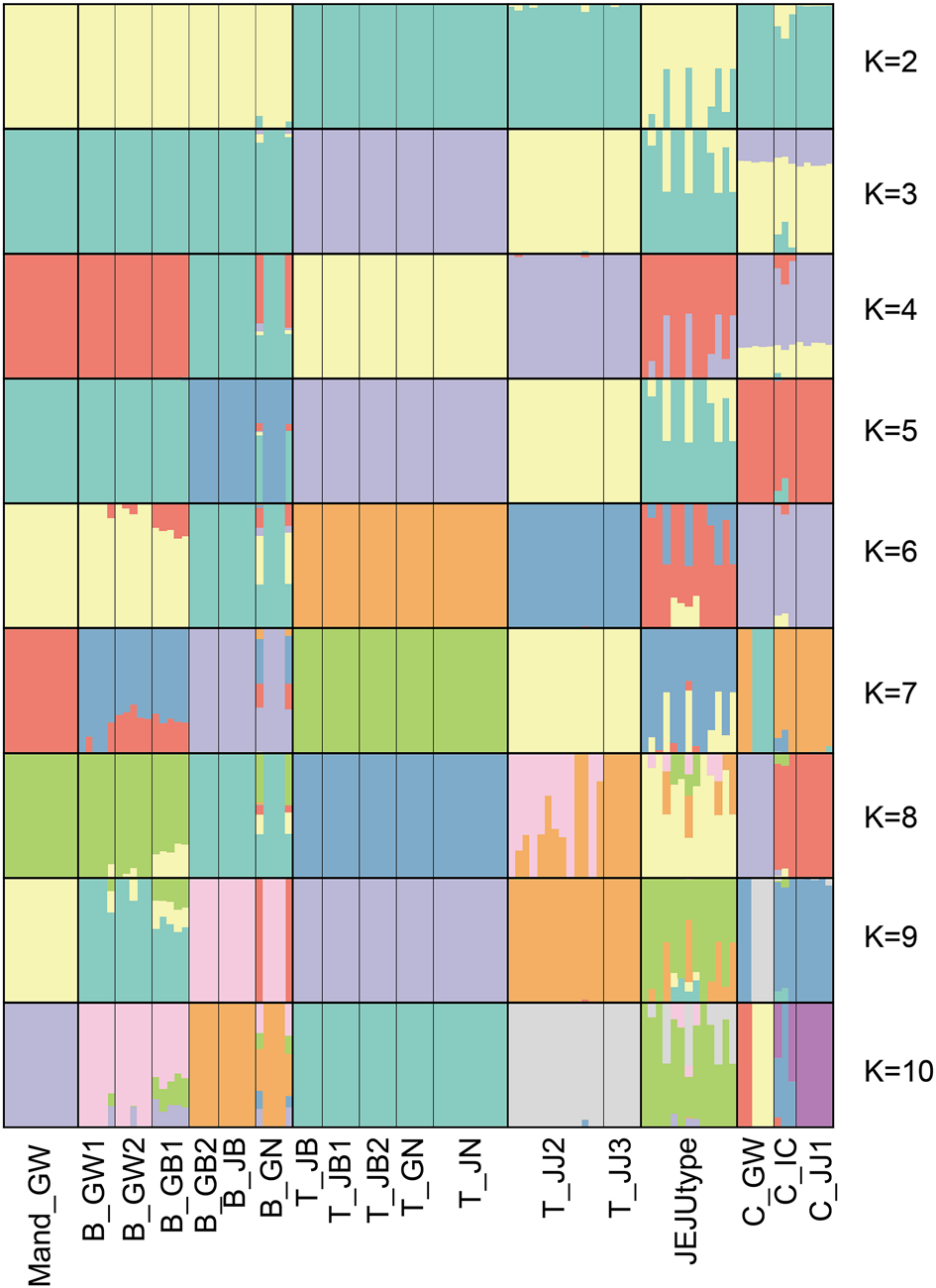
Genetic population structure prediction (K = 2–10). The distribution of the accessions to different populations is indicated by the color code.

Population genetic structure analysis (PCA and STRUCTURE) was conducted based on 8426 SNPs, using the STACKS v.2.60 software. The eigenvalues of PC1 (47.7%) and PC2 (11.35%) explained approximately 59.1% of the total variance (Fig. 4). PC1 separated the two major populations of wild *Malus* (excluding cultivar) from *M. toringo*, *M. baccata*, *M.* cf. *mandshurica*, and *M.* cf. *micromalus*. PC2 separated *M. toringo* into two groups, according to the geographic distribution. Three populations of cultivar were located in the center of *M. toringo* in PC2 (Fig. 4). From the assigned group, all samples clustered with each other except for *M.* cf. *micromalus* on JEJUtype population. JEJUtype accessions (hyh382, hyh386, hyh387, and hyh392) formed a group which was distinct from the other accessions in quadrant 4 (Fig. 4). This phenomenon was also observed in the STRUCTURE data (Fig. 5). We set the range of delta K as 2–10 for ancestral populations. Although K = 5 was determined as the optimal value from the CV error, the difference in CV values was insignificant; therefore, the results of K = 2–10 are presented (Fig. 5). *M. toringo* was divided into two distinct genotypic groups, one group with five populations (T_JB1, T_JB2, T_JB, T_GN, and T_JN) and the other with two populations (T_JJ2 and T_JJ3). The distinct groups remained consistent across the entire range of K values (Fig. 5). Two populations (T_JJ2 and T_JJ3) sampled from the southern Jeju Island of the Korean Peninsula showed a different genetic profile compared to that of the mainland *M. toringo* group (Fig. 5). Three accessions, including three cultivar populations (C_JJ1, T_GW, and T_IC) contained some of the mixed genetic compositions of *M. toringo*. Among the *M. baccata* populations, eight were divided into two clusters. The composition of the *M.* cf. *mandshurica* population revealed geographical similarities rather than species entity. The *M.* cf. *micromalus* (JEJUtype) population collected from Jeju Island shared both *M. toringo* and *M. baccata* genetic composition. Specifically, the *M. baccata* genotype was mixed with geographically more distant populations (B_GW1, B_GW2, and B_GB1) than closer populations (B_GB2, B_JB, and B_GN). The highly polymorphic and diverse JEJUtype population exhibited mixed components of *M. baccata* (B_GW1, B_GW2, and B_GB1) and *M. toringo* (T_JJ2 and T_JJ3).

### Population genetic diversity

**Figure 6:**
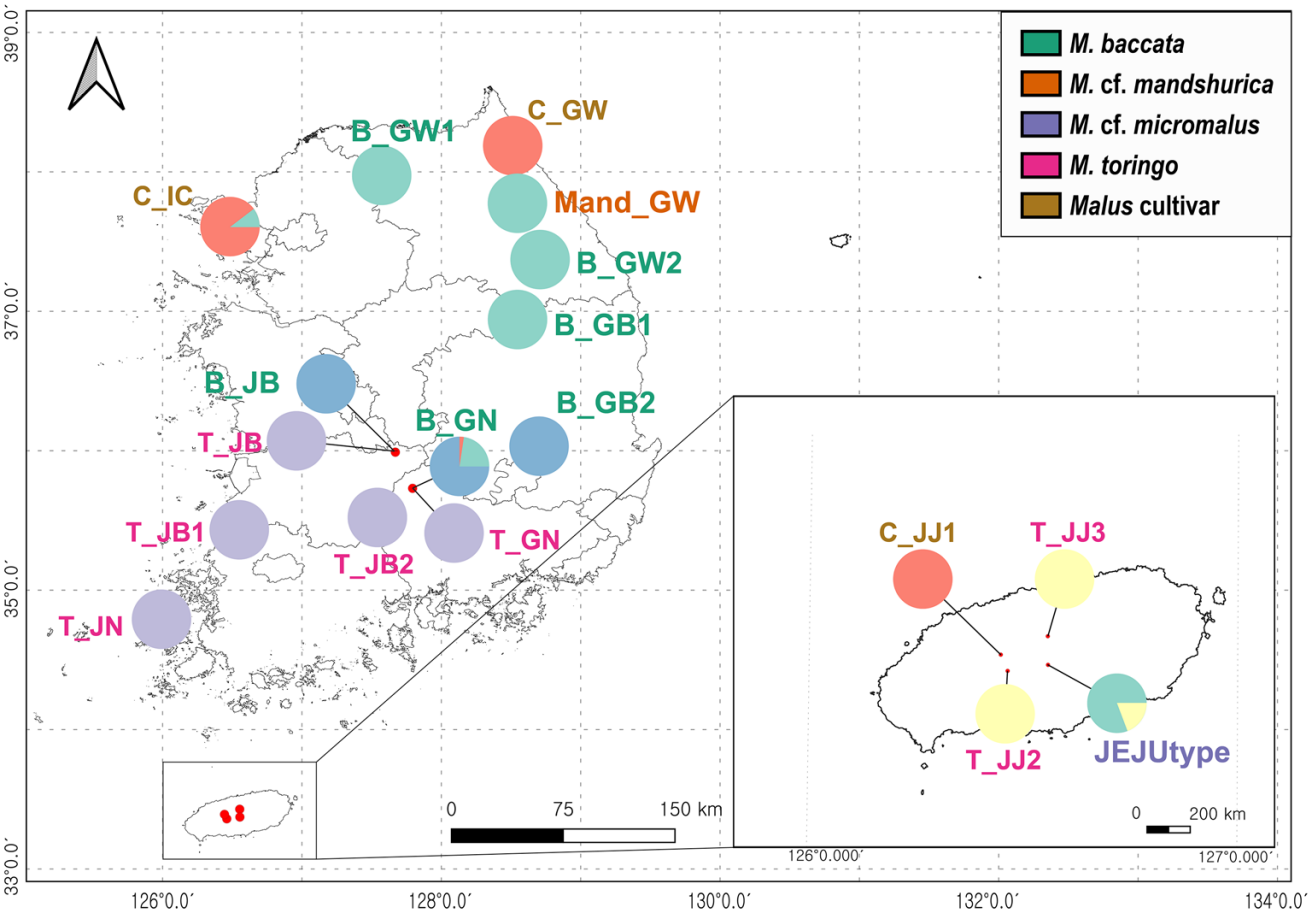
Pie charts of the location map used for the genetic population structure analysis of 18 *Malus* populations. The pie charts show the frequency of each cluster in a population based on STRUCTURE results (K = 5). Map source: https://www.gisdeveloper.co.kr/.

The mean expected heterozygosity (*He*) of the 18 *Malus* populations varied from 0.074 in T_GW to 0.203 in the island JEJUtype. The observed heterozygosity (*Ho*) of the 18 *Malus* populations varied from 0.074 in T_GW to 0.260 in B_GN. Wright’s F-statistic (*F*_*IS*_) of the 18 *Malus* populations ranged from -0.170 in B_JB to 0.042 in Mand_GW (Table 1). When the *Ho* value is higher than *He*, *F*_*IS*_ has a negative value, with most *Ho* values being higher than *He* (Table 1). The average nucleotide diversity (π) ranged from 0.083 in T_GW to 0.211 in the JEJUtype. High π was observed in three *M. baccata* (B_GB2, B_JB, and B_GN) groups, which were geographically distributed close to the *M. toringo* population (Fig. 6). The highest π was recorded in the *M.* cf. *micromalus* population from Jeju Island (Table 1).

### Genetic differentiation

Pairwise *F*_*ST*_ (genetic differentiation index) between populations ranged from 0.011–0.47 (Table 2); a similar trend was observed after the genetic structure analysis (Figs. 5 and 6). The lowest *F*_*ST*_ value was identified among *M. toringo* populations, specifically among five mainland groups (T_JB, T_JB1, T_JB2, T_GN, and T_JN) with similar geographical distributions. The lowest *F*_*ST*_ value was identified among the three *M. baccata* populations (B_GB2, B_GN, and B_JB). These findings indicated that the genetic difference within populations was greater than those between populations as well as the occurrence of gene flow between populations. Most *F*_*ST*_ values between the *M. baccata* and *M. mandshurica* groups as well as *M. toringo* were more than 0.3 (Table 2). *F*_*ST*_ values between JEJUtype, three *M. baccata* populations (B_GB1, B_GW1, and B_GW2), and three *M. toringo* populations (T_IC, T_JJ1, and T_JJ2) were extremely low. Additionally, the *F*_*ST*_ values between *M. mandsurica* and *M. baccata* (0.074–0.181) were lower or similar to the *F*_*ST*_ values measured between *M. baccata* populations, indicating the occurrence of gene flow between populations^69,70^.

**Table 2:** Pairwise genetic differentiation (*F*_*ST*_) values based on 8426 SNPs from 18 *Malus* population in Korean peninsula.

	C_GW	C_JJ1	C_IC	T_JB1	T_JB2	T_JJ2	T_JJ3	T_JB	T_GN	T_JN	B_GW1	B_GW2	B_GB1	B_GB2	B_JB	B_GN	JEJU type	Mand_GW
C_GW	0.00	0.12	0.15	0.29	0.29	0.16	0.23	0.29	0.29	0.28	0.44	0.42	0.39	0.42	0.41	0.34	0.19	0.35
C_JJ1		0.00	0.11	0.23	0.23	0.14	0.19	0.22	0.22	0.23	0.39	0.38	0.35	0.37	0.36	0.30	0.18	0.32
C_IC			0.00	0.23	0.23	0.14	0.19	0.23	0.23	0.23	0.33	0.33	0.30	0.31	0.31	0.25	0.12	0.27
T_JB1				0.00	0.01	0.22	0.29	0.01	0.01	0.02	0.47	0.45	0.42	0.45	0.44	0.36	0.22	0.38
T_JB2					0.00	0.22	0.30	0.01	0.01	0.02	0.47	0.45	0.42	0.45	0.44	0.36	0.22	0.38
T_JJ2						0.00	0.04	0.21	0.21	0.24	0.36	0.35	0.33	0.34	0.34	0.30	0.22	0.33
T_JJ3							0.00	0.29	0.29	0.30	0.41	0.40	0.37	0.39	0.39	0.33	0.20	0.34
T_JB								0.00	0.02	0.02	0.47	0.45	0.42	0.44	0.44	0.36	0.21	0.37
T_GN									0.00	0.02	0.47	0.45	0.42	0.45	0.44	0.36	0.22	0.38
T_JN										0.00	0.47	0.46	0.42	0.46	0.45	0.38	0.25	0.39
B_GW1											0.00	0.15	0.13	0.23	0.22	0.16	0.07	0.10
B_GW2												0.00	0.12	0.22	0.22	0.16	0.07	0.10
B_GB1													0.00	0.20	0.19	0.14	0.06	0.09
B_GB2														0.00	0.02	0.05	0.11	0.18
B_JB															0.00	0.05	0.10	0.18
B_GN																0.00	0.08	0.14
JEJU type																	0.00	0.07

C: *Malus* Cultivar, T: *M. toringo*, B: *M. baccata*, JEJUtype: putative *M. micromalus*, Mand: putative *M. mandshurica.*

### Molecular variance and migration rates

AMOVA results (Table 3) revealed that 52% of the genetic variation occurred between groups and 40% occurred within samples. Within samples, variation from the three measurements (*M. toringo*; *M. baccata*, and *M. mandshurica*; and *M. baccata*, *M. mandshurica*, and *M. micromalus*) was extremely high (84%, 96%, and 94%, respectively). Our analysis further revealed that between the two groups of *M. toringo* and *M. micromalus*, 52% of the total variations occurred within samples, while the remaining 37.8% and 19.2% of variations occurred between groups and between populations/within group, respectively. The results of the migration rate analysis revealed that the Nm between all populations without model specification was 0.373, with the highest value obtained (0.748) at M3 → 5 (Table S4).

**Table 3:** Summary on the analyses of molecular variance (AMOVA) in *Malus* and its relatives across 8426 SNPs, showing degree of freedom (df), sum of squares (SS), variance components, and the total variance contributed by each component (%) and its P value. Asterisked scientific name included SRA and collected data.

Taxon (species)	Source of variation	Df	SS	Variance components	Total variance (%)	P value
ToTal (112)	Between group	3	158,847.73	959.498	40.239	0.01
	Between pop within group	14	72,792.03	403.034	16.902	1
	Between samples Within pop	94	73,428.15	− 240.803	− 10.099	0.01
	Within samples	112	141,428.68	1262.756	52.957	0.01
*M. toringo* (60)	Between pop	9	52,259.57	448.892	35.652	0.01
	Between samples Within pop	50	27,978.32	− 250.645	− 19.907	1
	Within samples	60	63,651.38	1060.856	84.255	0.01
*M. baccata* and M. mandshurica* (39)	Between group	1	6921.8	4.194	0.284	0.46
	Between pop within group	5	20,759.23	343.087	23.196	1
	Between samples Within pop	32	26,857.15	− 292.537	− 19.778	0.01
	Within samples	39	55,550.04	1424.360	96.299	0.41
*M. baccata*, M. mandshurica* and M. micromalus* (52)	Between group	2	14,453.81	− 7.417	− 0.469	0.2
	Between pop within group	5	20,691.25	321.247	20.321	1
	Between samples Within pop	44	45,608.43	− 230.503	− 14.581	0.01
	Within samples	52	77,873.17	1497.561	94.729	0.58
*M. toringo* and M. micromalus* (73)	Between group	1	47,386.42	848.931	37.805	0.01
	Between pop within group	9	52,137.06	431.154	19.200	1
	Between samples Within pop	62	46,704.53	− 212.161	− 9.448	0.01
	Within samples		73	1177.621	52.443	0.09

## Discussion

Although previous studies have examined the genetic diversity of *Malus* in Europe^71–73^ and central Asia^74,75^, with several reports on cultivar^76,77^, studies on *Malus* in the Korean Peninsula are limited. In this study, we evaluated the genetic diversity and structure of *Malus* species in the East Asia, using a GBS-based analysis. The molecular diagnosis used in this study provides extensive information on variations of the Korean *Malus*, which could facilitate the tracking of geneflow, and deciphering of taxonomic delimitation, and historical evolution of divergence.

The level of heterozygosity was significantly lower within the Korean *Malus* population (mean *He* = 0.07–0.2; mean *Ho* = 0.07–0.26; Table 1) compared with that reported in previous studies (*He* > 0.6; *Ho* > 0.7)^71,72,78^. However, this discrepancy could be attributed to differences in research methodology^79^. Moreover, the findings of the present study are in agreement with those of previous GBS-based studies^80^. However, the low diversity observed in most Korean *Malus* populations is unusual for *Malus* (Table 1), which is self-incompatible and relies on cross pollination^81^. Other evolutionary forces affecting genetic diversity include mode of reproduction, geographical distribution, and population size, among which the mode of reproduction is highly relevant as it involves direct genetic exchange^82^.

The *F*_*IS*_ values obtained in this study were close to zero in six populations and negative in the remaining ones (Table 1). Also, the *F*_*IS*_ values show regional differences even within the same species (Table 1 and Fig. 6). For instance, the *F*_*IS*_ value of *M. baccata* converges to 0 and − 0.1 in C1 and C2, respectively (Table 1). Generally, outcrossing species are known to exhibit a higher genetic diversity than that of selfing species^83^; nevertheless, it is surprising that the genetic diversity in our results is low in *Malus* (*He*, *Ho,* and Pi; Table 1). It is possible that the low genetic diversity is a result of spatial isolation^80^, or that *Malus,* specifically in the Korean Peninsula, has high homozygosity. Geographical barriers and small population sizes facilitate genetic drift and bottlenecks^84,85^. Geographically, the Korean Peninsula is surrounded by the sea along the east, west, and south coasts which can limit gene flow into the Korean *Malus* population from marginal states. Such spatial or landscape structure barriers can increase genetic isolation^80^.

### Molecular diagnosis of *M. baccata* and *M. toringo* distributed in the Korean Peninsula

*M. baccata* and *M. toringo* from the Korean Peninsula can be differentiated and described based on distinct morphological features, such as leaf lobes, length of petioles, pedicel, fruit size, and bud character^11–14,25–27,34^. However, morphologically intermediate species from geographically overlapping regions have been reported^27^. Contrary to our hypothesis that hybridization would be observed, our results indicated the presence of two genetically distinct groups, *M. baccata* and *M. toringo,* without hybrid signals (Figs. 2, 3, 4, and 5). From K = 4–7, the genetic structure between *M. baccata* and *M. toringo* was clearly distinguished, and no indication of hybridization between the two species was detected in the mainland population (Figs. 5 and 6). In B_GN, some individuals showed mixed profiles that were affected by cultivar genotypes of individuals planted in the recreation forest (Fig. 5). Additionally, traces of genetic exchange between JB and GN have not been identified in this study despite the similar forest distribution of the two species, implying reproductive isolation between the two species (Fig. 6). PCA indicated that *M. baccata* and *M. toringo* were clearly distinguished based on the PC1 component (Fig. 4). Moreover, most *M. baccata* populations share similar genetic profiles; the mainland *M. toringo* was clearly distinguishable from the Jeju Island group based on the PC2 component (Fig. 4).

Notably, it is important to understand the distribution and spatial pattern of living organisms to interpret their biological differentiation and ecological evolutionary history^86^. The segregation of biogeographic regions is based on geography, geology, and climate^87^. Several studies attempted to subdivide the biogeographic region of the Korean Peninsula^88–91^. Jung and Cho^91^ segregated four biogeographic regions based on the coordinates of 310,000 vascular plant specimens collected from the Korean Peninsula. In the population groups redefined in Table 1, C1 and C7, which share similar genetic profiles (Fig. 6) belonged to the central Korean Peninsula, named Zone I (Cold floristic zone) and Zone II (Cool floristic zone)^91^. C3, which included five populations of *M. toringo* with similar genetic profiles belonged to Zone III (Warm floristic zone) and Zone IV (Miratic zone 2)^91^. C2 belonged to the boundary between Zone II and Zone III in the low-altitude regions of the Korean Peninsula^91^, which is similar to the biogeographic regional boundary proposed by Lee and Yim^90^. Nevertheless, the possibility that the distance between populations, altitude, and climatic zone changes affect the genetic composition of *Malus* cannot be ruled out (Table 2).

PCA and STRUCTURE analysis results indicated the segregation of *M. toringo* into two distinct groups: mainland and Jeju Island populations (Figs. 4, 5, 6). Additionally, the 35.65% difference “between pop” of *M. toringo* populations in the molecular variation analysis suggests that the analyzed individuals could belong to more than one group (Table 3). With an increase in the K value, the difference between the two groups remained distinct, suggesting that gene flow was not a recent occurrence (Fig. 5). A historical migration rate of 0.748 was observed between the mainland *M. toringo* (C3 in Table 1) and Jeju Island *M. toringo* (C5 in Table 1 and Table S4). Genetic variations resulting from mutation, recombination, and division of basic gene pool can cause the emergence of a morphologically or genetically evolutionarily significant units^82,92^. Our results imply *M. toringo* is genetically difference groups between mainland (C3 in Table 1) and Jeju Island (C5). However, our data have limitation for verifying the evolutionarily significant units as species. Therefore, further studies in aspect of morphological and molecular methods are necessary with extensive sampling.

### *M.* cf. *micromalus* on Jeju Island

Interspecific hybridization of *Malus* has been inferred in the past^7^ and confirmed by a recently conducted phylogenomic analysis of *Malus* hybridization^93^. The genotypes of symmetric hybrids are composed of half of each putative parent genotype^94^ and share similar structural patterns with the cultivar cluster identified in this study (Fig. 5). It has been hypothesized that *M. micromalus* is a hybrid of *M. baccata* and *M. spectabilis* (as detailed in the “Introduction”); however, this has never been evaluated. The *M.* cf. *micromalus* population (JEJUtype) collected from Jeju Island did not exhibit a close relationship with SRA-*M. micromalus*, nor did it show a close relationship with *M. spectabilis* (Fig. 2A). Some individuals within the JEJUtype clustered with *M. baccata* while others were positioned between the *M. baccata* and *M. toringo* lineage (Fig. 2A).

**Figure 7:**
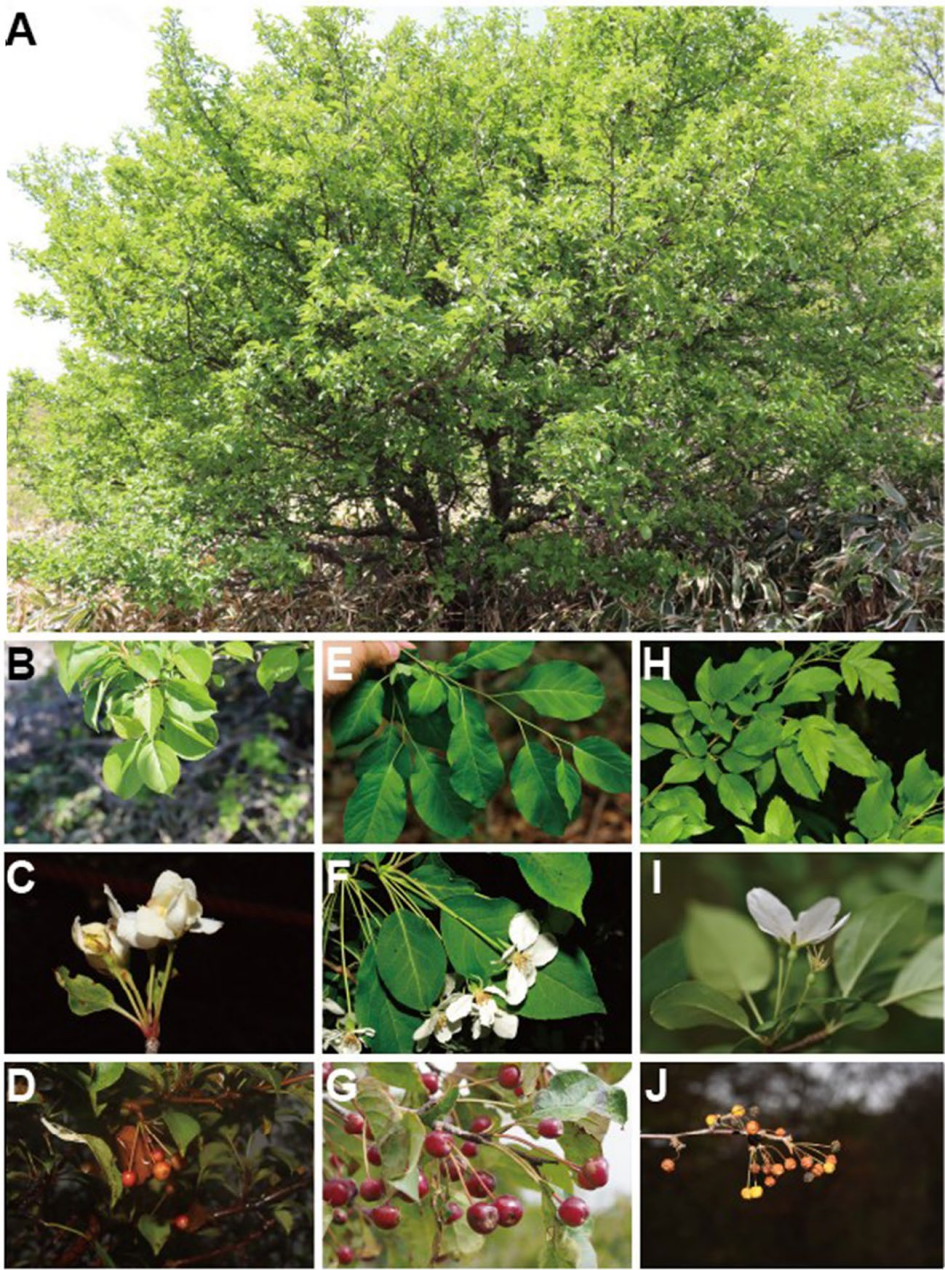
Photos of habitat, leaf, inflorescence, and fruits of *Malus* in the Korea Peninsula: A–D: *M. micromalus* (in Jeju Island). E–G: *M. Baccata* (in GW). H–J: *M. Toringo* (in JB). All photograph was taken by Y.-H.H.

The present study indicated that the JEJUtype did not cluster with previously reported targets (*M. micromalus*; Fig. 2) and should be treated as a separate lineages generated by hybridization between C5 (JJ2 and JJ3) and *M. baccata* (Fig. 5)^95,96^. Additionally, one-sided genetic invasion from C5 to *M. baccata* is presumed to be the result of asymmetric hybridization, where C5 acts as a pollen donor and *M. baccata* as a pollen acceptor (Fig. 5)^97–99^. Only a few JEJUtype individuals were identified as F1 descendants with half the genotypes of both parental species (Fig. 5). A similar case has been reported in association with *Prunus yedoensis* in Rosaceae^100^. JEJUtype species are characterized by lanceolate-ovate leaves similar to those of *M. baccata* and short peduncles similar to those of *M. toringo*; the species are also characteristic of small-sized, narrow, and unlobed leaves (Fig. 7). The major taxonomical key character of *M. micromalus*, the persistence of sepal in fruit, was not identified in this population. Based on these findings, we suggest that JEJUtype (*Malus* cf.), which has been the center of debate for nearly 100 years since Makino described a new species (*M. micromalus*) in 1908^33^, is a different entity from *M. micromalus*. To better understand the evolutionary history of this hybrid, extensive sampling is necessary to identify genetic clues in their natural habitat and assess their phenetic characters, particularly in the species-rich neighboring countries (China and Japan).

### Taxonomical decision of *M. mandshurica*

The *M.* cf. *mandshurica* (C7) analyzed in this study exhibited similar morphological characteristics with previously identified *M. mandshurica*, such as tomentose hair on petiole and adaxial leaf pubescence^25,26^. However, SplitsTree analysis indicated that there was no close relationship between *M.* cf. *mandshurica* accessions (population: Mand_GW) and SRA-*M. mandshurica*, which is a remarkable finding (Fig. 2). Additionally, the analyses conducted in this study indicated that C7 shares a similar genetic profile with *M. baccata* (Figs. 4 and 5; Tables 2 and 3). However, previous studies have reported inconsistencies in the identification/characterization of *M. mandshurica*, with some studies reporting a close relationship between *M. mandshurica* and *M. baccata* or *M. micromalus*^28,29,31,37^. Physiologically, studies have reported that the leaves of plants become smaller and thicker, and the amount of hair increases as the altitude increases^101,102^; thus, further studies are needed to analyze the status of *M. mandshurica* at the species level.

## Conclusion

The current study primarily explored the genetic structure and geneflow of *Malus* in the Korean Peninsula using GBS analysis. Following sequencing, we identified high quality SNPs (8426) using the reference mapping method. Notably, majority of the *Malus* populations distributed in the Korean Peninsula form geographically distinct groups that coincide with those in the floristic zones^91^. Contrary to our prediction that hybridization would occur between *M. baccata* and *M. toringo*, we observed that the two species were genetically differentiated. Putative *M. micromalus* from the Jeju Island might represent a new hybrid. Our findings provide valuable insights into the genetic profile of Korean *Malus*. However, thorough morphological studies with extensive sampling are needed to clarify these species attributes.

## Supplementary Information

Supplementary Tables.

## Data Availability

All sequencing data analyzed in this study are publicly available from the National center of Biotechnology Information (https://www.ncbi.nlm.nih.gov/) under the BioProject ID: PRJNA826537.
